# Residential proximity to active and abandoned oil and gas development and risk of childhood Ewing sarcoma in California

**DOI:** 10.1186/s12940-025-01259-3

**Published:** 2026-01-14

**Authors:** Cassandra J. Clark, Nicholaus Johnson, Rong Wang, Eric C. Stewart, Logan G. Spector, Joseph L. Wiemels, Catherine Metayer, Nicole C. Deziel, Xiaomei Ma

**Affiliations:** 1 Division of Pediatric Epidemiology and Clinical Research, Department of Pediatrics, University of Minnesota School of Medicine, Minneapolis, MN USA; 2 Department of Environmental Health Sciences, Yale School of Public Health, New Haven, CT USA; 3 Department of Chronic Disease Epidemiology, Yale School of Public Health, New Haven, CT USA; 4 Division of Epidemiology, School of Public Health, University of California, Berkeley, CA USA; 5 Center for Genetic Epidemiology, Department of Population and Public Health Sciences, University of Southern California, Los Angeles, CA USA; 6 Yale Comprehensive Cancer Center, Yale School of Medicine, New Haven, CT USA

## Abstract

**Background:**

Oil and gas development (OGD) has been linked to increased pediatric cancer risk, but the literature to date is focused on hematologic malignancies and active wells. The emergence of suspected clusters of cancers such as Ewing sarcoma in children living near OGD and widespread presence of abandoned wells warrants investigation.

**Methods:**

This study included 558 children born in California (1982–2015) reported to the California Cancer Registry with a diagnosis of Ewing sarcoma at 0–19 years (1988–2015), and 27,800 cancer-free controls frequency-matched to cases on birth-year (50:1 ratio). We used birth address to assign prenatal OGD exposure to active (drilled or producing) and plugged/abandoned wells separately with inverse distance-squared weighted well counts at 5 and 10 km buffer sizes from three months before conception to birth. We evaluated potential exposure disparities and estimated odds ratios (OR) and 95% confidence intervals (CI) for the association between prenatal OGD exposure and Ewing sarcoma risk using multivariable logistic regression.

**Results:**

Hispanic children were significantly more likely to be exposed to both active and abandoned OGD within 10 km than non-Hispanics (40% vs. 23% and 14% vs. 6%, respectively). There were no associations between prenatal exposure to active OGD within 10 km and Ewing sarcoma risk (OR: 0.88 [95% CI: 0.72–1.08]). However, children within 10 km of abandoned wells were 1.27 [0.96–1.66] times as likely to develop Ewing sarcoma as unexposed children; when stratified by ethnicity, this association appeared in Hispanic children only (1.33 [0.95–1.88]).

**Conclusion:**

We did not identify an association between exposure to active OGD and pediatric Ewing sarcoma risk in California. Abandoned wells were associated with a suggestive increase in risk among Hispanic children, who were also more likely to be exposed to any OGD activity than non-Hispanic children. This disparity could have implications for other health outcomes including childhood cancers.

**Supplementary Information:**

The online version contains supplementary material available at 10.1186/s12940-025-01259-3.

## Introduction

Ewing sarcoma is a rare, aggressive cancer of the bones and soft tissues occurring primarily in children and adolescents (median age of onset: 14–15 years) [1] and most commonly among children of European ancestry [2, 3]. While the prognosis for children in whom the disease is localized is fair (5-year survival: 65–80%), once metastasis occurs the survival rate drops dramatically (5-year survival: <30%)[3–6] The incidence of Ewing sarcoma has been increasing in North America, particularly in younger children (1.79% average annual change for the 0–9 year age group from 1988–2012) [7].

Current understanding of the etiology of Ewing sarcoma is limited [8–10]. The development of Ewing sarcoma generally results from chromosomal translocations between the *EWSR1* gene and a member of the *ETS*family of genes[3, 8, 11], most commonly *FLI-1*. The resulting EWS-FLI1 fusion protein binds to GGAA microsatellites in target genes[12], and can enhance oncogenesis by interfering with normal cellular activities, such as cell cycle regulation and differentiation [8–14]. Evidence from cell models suggests that the presence of EWS-FLI1 alone (present in 85% of cases) may not be sufficient to cause Ewing sarcoma, and that co-occurring mutations or modifications are necessary [15–17]. Environmental exposures could provide a mechanism for such co-occurring mutations – and even the formation of the translocation itself.

No specific environmental risk factors for Ewing sarcoma have been identified to date, and Ewing sarcoma is underrepresented in the environmental literature to date. There have been a few investigations into the role of geospatially varying environmental risk factors in the occurrence of potential clusters of Ewing sarcoma [18, 19]. Pesticides[20–25], proximity to industrial activity (particularly to those industries releasing polyaromatic hydrocarbons)[26], and air pollution [27] have all been implicated. Occupational parental exposures and exposures during pregnancy have also been linked to Ewing sarcoma risk, which suggests a potential prenatal or early life origin [19, 21]. The results of these studies tend to be mixed, perhaps in part due to the rarity of Ewing sarcoma limiting the statistical power.

Oil and gas development (OGD), which uses and releases carcinogenic compounds[28–32], is another potential risk factor. OGD has been linked to increased childhood leukemia risk[33–37], but its potential influence on risk of other pediatric cancers remains understudied. Additionally, more than 4.6 million people live within 1 km of one of the 2.3 million to more than 3 million documented plugged or abandoned wells in the United States[38]; little is known about the potential emissions or health effects associated with these wells [39–44]. The emergence of suspected clusters of other cancers such as Ewing sarcoma in children living near active and abandoned OGD warrants investigation [5, 6]. 

Despite public concern about potential clusters of rare childhood cancers like Ewing sarcoma arising near OGD, the literature on this topic is sparse and focused primarily on leukemia [28, 33, 34, 45–48]. The single peer-reviewed study evaluating Ewing sarcoma risk in the context of OGD exposure examined a small number of cases (*n* = 20). Additionally, most OGD research to date has focused on only active wells rather than abandoned/plugged wells, which may emit similar contaminants [40]. To better understand patterns of exposure to both active and abandoned OGD wells and their impacts on an understudied, rare cancer we conducted a population-based case-control study of prenatal exposure to OGD and risk of childhood Ewing sarcoma in California, the most populous state in the United States.

## Methods

### Study population and data sources

Our study population is drawn from the California Linkage Study of Early-onset Cancers (CALSEC), a linkage of cancer diagnoses (age 0–39 years) reported to the California Cancer Registry from 1988 to 2015 and statewide birth records from 1978 to 2015. CALSEC also includes millions of controls who were born in California and had not been diagnosed with any cancer at the age of 0–39 years by 2015 based on information from the California Cancer Registry. Our initial study sample included a total of 619 children who were born in California during 1978–2015 and diagnosed with first primary and malignant Ewing sarcoma at the age of 0–19 years during 1988–2015 (i.e., cases), as well as 30,950 control subjects frequency-matched to cases on year of birth at a 50:1 ratio. After exclusions for missing residential address information or inadequate geocode quality (*n* = 61 cases and 3,150 matched controls, most born during 1978–1981, when only ZIP code level information was available) the final study sample consists of 558 cases and 27,800 birth year-matched controls.

Individual level covariates (e.g., sex, race, ethnicity, birth weight) were abstracted directly from birth records. For socio-economic status (SES) at the census tract level, we used the Centers for Disease Control/Agency for Toxic Substances and Disease Registry Social Vulnerability Index (SVI)[49], a composite index representing sixteen different factors related to socio-economic status, household characteristics, housing type and transportation, and racial and ethnic minority status. The SVI is represented as a percentile (0 to 1.0), with increasing values corresponding with increasing vulnerability. The covariates assessed as potential confounders were selected a priori based upon the literature and included sex, maternal race and ethnicity, birth weight, and socio-economic status [2, 50–53]. The study protocol has been approved by the Institutional Review Boards at the California Health and Human Services, University of Southern California, and Yale University.

### Exposure assessment

Oil and gas data were collected from Enverus’s Drilling Info platform on March 26, 2024. Data retrieved included all California wells in the “Wells” database (*n* = 253,171). We restricted our exposure dataset to directionally, horizontally, and vertically drilled oil and gas wells spud (i.e., drilled) or producing during the period of interest (1980–2015) with a status of ‘active’ or ‘plugged and abandoned’. Eligible wells were those that were documented as (a) active (drilled or producing) or (b) plugged or abandoned during the exposure window of interest. For wells with a first production start date but no spud date, we assigned a spud date by subtracting the median number of days between spud and first production, from the first production date. “Active” status includes wells that have never been designated as “Plugged and Abandoned”; only the active time window was considered for these wells. “Plugged and abandoned” status includes wells that have been designated as “Plugged and Abandoned”; only the plugged and abandoned time window was considered, defined as the day after the last production date through the end of the study period. Because many plugged and abandoned wells lacked sufficient information to assign them cutoff dates, we focused on those wells clearly designated as ‘plugged and abandoned’ during our study period. This data was further cleaned to remove duplicates, resolve missing data, and fix structural errors.

We used maternal residential address at the time of birth, obtained from birth records and geocoded by the University of California Berkeley[54], to assign OGD exposure metrics individually for each case and control during the prenatal exposure window of interest, three months before conception to birth. The critical window of exposure for Ewing sarcoma is poorly understood. We chose to focus on the prenatal period both because many childhood cancers are thought to initiate prenatally [50, 55, 56] and because of the association between congenital hernia and Ewing sarcoma risk[57, 58], which also supports a potential prenatal origin. To represent OGD exposure for each subject, we calculated (1) distance to nearest well, (2) number of wells within a buffer zone, and (3) an inverse distance-squared weighted (ID^2^W) well count. The ID^2^W metric was constructed using the Euclidean distance between the subject’s home and surrounding eligible OGD wells within 1, 2, 5, and 10 km (ArcGIS 10.8.1). All distances are Euclidian using a projected coordinate system specific to California (EPSG:3310). We selected these buffer sizes based on the hydrogeologic (supports contaminant transport distances of 2 km or less [30, 59, 60]) and epidemiologic (incorporates buffer distances up to 20 km[61, 62]) literature. Exposure metrics were averaged over the primary prenatal exposure window, three months before conception to birth, to estimate cumulative exposure. OGD exposures were operationalized as ordinal (for number of wells within a buffer zone), binary (any or no exposure), and as tertiles (low/medium/high exposure). All distance calculations were completed using the latest version of R (R version 4.4.0 as of June 2025).

### Statistical analysis

All statistical analyses were conducted in SAS 9.4, and all tests were two-sided with an alpha level of 0.05. We used chi-square and t-tests to identify differences in the distribution of population characteristics and OGD exposure between case and control children. We used unconditional logistic regression to estimate odds ratios (OR) and 95% confidence intervals (CI) for the association between active, plugged and abandoned, and all OGD exposure and risk of pediatric Ewing sarcoma, adjusting for year of birth (the matching variable), sex, race, ethnicity, birth weight (continuous, per 500 g) and community-level SES.

### Sensitivity analyses

Because people of color are more likely to be disproportionately exposed to environmental hazards including OGD in the United States[63–70], we compared exposure distributions and stratified models by race and ethnicity when possible to evaluate potential exposure inequalities with respect to OGD. Rurality is an important predictor of OGD siting [71]. Additionally, we conducted a sensitivity analysis using unconditional logistic regression to estimate the association between OGD exposure and risk of Ewing sarcoma adjusting for region of birth (assigned by County) in addition to all other confounders included in the main model (year of birth [the matching variable], sex, race, ethnicity, birth weight [continuous, per 500 g] and community-level SES).

## Results

### Population characteristics

Case children were more likely to be male (58% vs. 51%, *p* < 0.01), have a birth weight > 4000 g (14% vs. 11%, *p* = 0.01), and be non-Hispanic White (48% vs. 37%, *p* < 0.01; Table 1) than control children. Case children also had significantly lower social vulnerability indices than control children across all domains examined, indicating lower social vulnerability and higher SES (all *p*≤0.02; Table 2). When stratified by Hispanic ethnicity, this pattern remained evident among non-Hispanic cases, but not among Hispanic cases (Table 2). Most of the cases included in this study were diagnosed at 10–14 (33%) and 15–19 (29%) years of age.

**Table 1:** Characteristics of the study population

Characteristic	Cases (*n* = 558)	Controls (*n* = 27,800)	*p*-value*
Sex	N (%)	N (%)	
Male	326 (58)	14,185 (51)	< 0.01
Female	232 (42)	13,615 (49)	
Age at diagnosis (years)			
0–4	130 (23)	-	
5–9	81 (15)	-	
10–14	183 (33)	-	
15–19	164 (29)	-	
Gestational age (weeks)			0.21
< 32	2 (1)	423 (1)	
32 - <37	40 (7)	2192 (8)	
37 - <39	105 (19)	5348 (19)	
39–41	318 (57)	15,203 (55)	
≥ 42	93 (17)	4639 (17)	
Birth weight (grams)			0.01
< 1500	0 (0)	297 (1)	
1500–2499	25 (5)	1418 (5)	
2500–3999	455 (82)	22,977 (83)	
≥ 4000	78 (14)	3077 (11)	
Mode of delivery			0.93
Vaginal	436 (78)	21,681 (78)	
Cesarean	122 (22)	6124 (22)	
Race and ethnicity			< 0.01
Non-Hispanic White	268 (48)	10,222 (37)	
Non-Hispanic Black	4 (1)	2259 (8)	
Hispanic	239 (43)	12,394 (45)	
Non-Hispanic Asian or Pacific Islander	41 (7)	2598 (9)	
Other or Unknown Race	6 (2)	332 (2)	
Maternal age (years)			0.22
< 20	57 (10)	3314 (12)	
20–24	134 (24)	7364 (26)	
25–29	167 (30)	8065 (29)	
30–34	139 (25)	5978 (22)	
≥ 35	61 (11)	3084 (11)	
Mother’s educational attainment			0.10
8th grade or less	41 (7)	2511 (9)	
9th − 12th grade	156 (28)	8365 (30)	
Some college or more	153 (27)	6530 (23)	
Unknown	208 (37)	10,399 (37)	
	Mean (SD)	Mean (SD)	
Percent of block group with < high school education	30.73 (21.48)	31.40 (20.70)	0.49
Percent of block group with ≥college education	47.07 (21.05)	45.85 (20.24)	0.20
Percent of block group in poverty	26.15 (19.17)	27.61 (19.04)	0.10

Categorical variables in Table 1 were presented as frequencies and percentages, with p values derived from *Chi-square or Fisher’s Exact tests. The following variables were presented with means, standard deviations (SD) and p values derived from t-tests: percent of block group with different levels education attainment or in poverty, social vulnerability index

**Table 2:** Distribution of social vulnerability indices, stratified by Hispanic ethnicity

Characteristic	Full population			Non-Hispanic			Hispanic		
	Cases (*n* = 558)	Controls (*n* = 27,800)	*p*-value	Cases (*n* = 255)	Controls (*n* = 12,369)	*p*-value	Cases (*n* = 212)	Controls (*n* = 10,899)	*p*-value
	Mean (SD)	Mean (SD)		Mean (SD)	Mean (SD)		Mean (SD)	Mean (SD)	
Social Vulnerability Index (percentile)	0.58 (0.29)	0.62 (0.28)	< 0.01	0.45 (0.27)	0.54 (0.28)	< 0.01	0.73 (0.24)	0.72 (0.24)	0.55
Socio-economic domain	0.57 (0.29)	0.60 (0.28)	0.01	0.44 (0.27)	0.51 (0.28)	< 0.01	0.72 (0.24)	0.70 (0.25)	0.17
Housing and transportation	0.58 (0.29)	0.63 (0.27)	< 0.01	0.50 (0.28)	0.57 (0.28)	< 0.01	0.69 (0.26)	0.68 (0.25)	0.93
Household composition and disability	0.56 (0.29)	0.59 (0.28)	0.02	0.47 (0.29)	0.53 (0.30)	< 0.01	0.68 (0.25)	0.67 (0.25)	0.61
Minority status and language	0.56 (0.30)	0.61 (0.28)	< 0.01	0.42 (0.27)	0.51 (0.27)	< 0.01	0.72 (0.24)	0.72 (0.24)	0.96

*P*-values derived from t-test

### Oil and gas exposure distribution

**Fig. 1:**
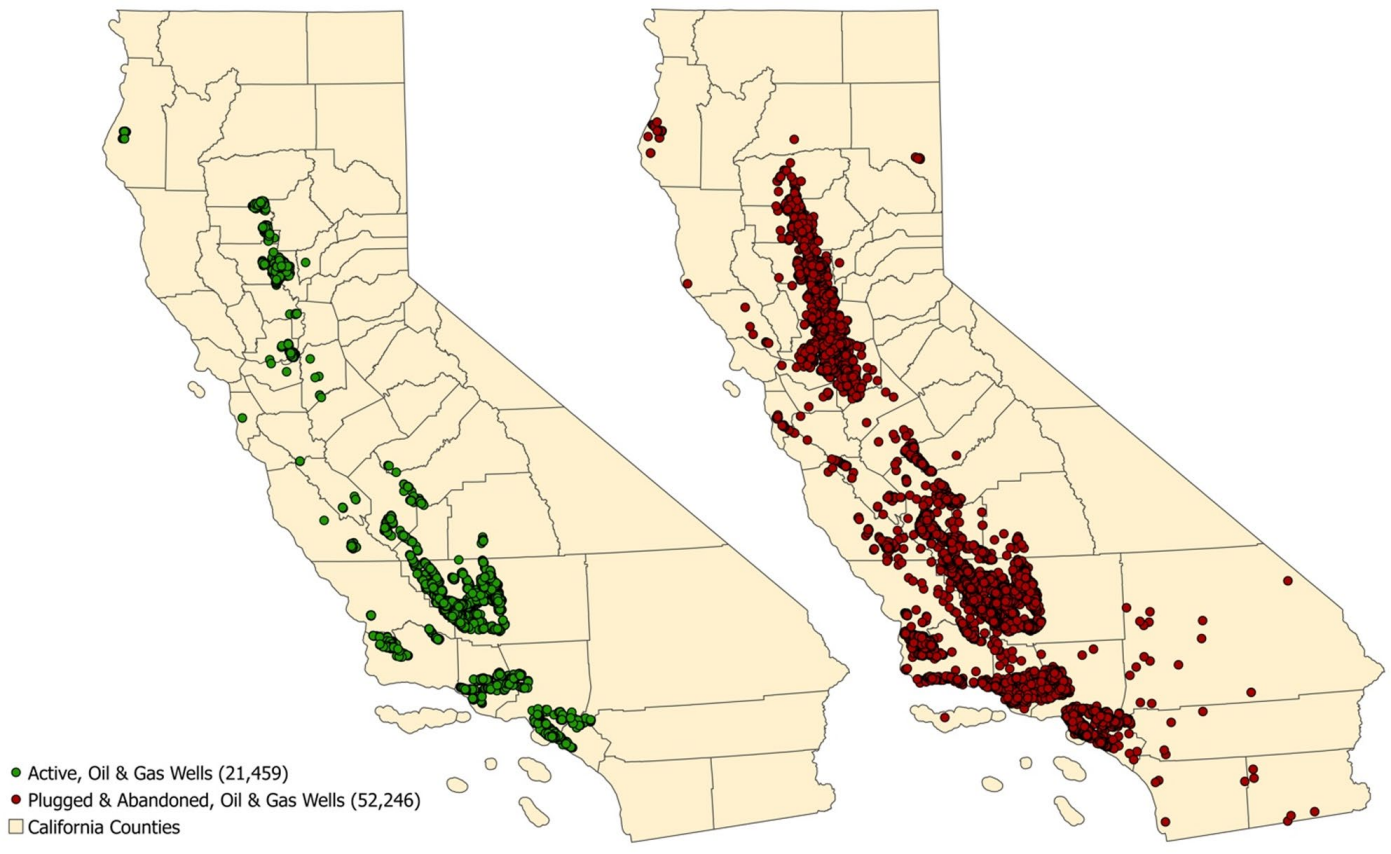
Active (green) and plugged/abandoned (red) oil and gas wells in California during the study period (1980–2015)

There were *n* = 21,459 and 52,246 active and abandoned/plugged OGD wells in California, respectively, during the study period (Fig. 1). Approximately 17% and 27% of cases and 19% and 31% of controls had an active OGD well within 5–10 km of the birth residence, respectively (Table 3). Exposure to abandoned wells was less common, with 11% of cases and controls having an abandoned well within 10 km of the birth residence. Notably, significantly more Hispanic cases were exposed to any active or abandoned OGD as compared to non-Hispanic cases (all *p* < 0.01; Table 3). For instance, 38% of Hispanic cases had at least one active well within 10 km of the birth residence as compared to 19% of non-Hispanic cases (*p* < 0.01). This was also true in the combined population of cases and controls. However, when both proximity and density of wells was considered, non-Hispanic cases and controls tended to be more highly exposed. For instance, 49% of non-Hispanic cases fell into the highest tertile of active OGD activity within 10 km as compared to 27% of Hispanic cases (*p* < 0.01).

**Table 3:** OGD exposure distribution among case and control children, stratified by Hispanic ethnicity

Exposure	Total population			Stratified by Hispanic ethnicity					*p*-value
	Cases (*n* = 558)	Controls (*n* = 27,800)	*p*-value	Non-Hispanic cases (*n* = 319)	Hispanic cases (*n* = 238)		Non-Hispanic cases and controls, combined (*n* = 15,727)	Hispanic cases and controls, combined (*n* = 12,629)	
	*N* (%)	*N* (%)		*N* (%)	*N* (%)		*N* (%)	*N* (%)	
Any active wells within 5 km			0.14			< 0.01			< 0.01
Yes	94 (17)	5375 (19)		39 (12)	55 (23)		2217 (14)	3252 (26)	
No	464 (83)	22,430 (81)		280 (88)	184 (77)		13,513 (86)	9381 (74)	
Any active wells within 10 km			0.06			< 0.01			< 0.01
Yes	150 (27)	8502 (31)		59 (19)	91 (38)		3569 (23)	5083 (40)	
No	408 (73)	19,303 (69)		260 (81)	148 (62)		12,161 (77)	7550 (60)	
Any abandoned wells within 5 km			0.53			< 0.01			< 0.01
Yes	33 (6)	1476 (5)		10 (3)	23 (10)		542 (3)	967 (8)	
No	525 (94)	26,329 (95)		309 (97)	216 (90)		15,188 (97)	11,666 (92)	
Any abandoned wells within 10 km			0.22			< 0.01			< 0.01
Yes	62 (11)	2656 (11)		20 (6)	42 (18)		972 (6)	1746 (14)	
No	496 (89)	25,149 (89)		299 (94)	197 (82)		14,758 (94)	10,887 (86)	

Inverse distance-squared weighted well count	N (%)	N (%)	p-value	N (%)	N (%)		N (%)	N (%)	p-value
Active wells									
5 km			0.63			< 0.01			< 0.01
Unexposed	462	22,425		279	183		13,510	9377	
Tertile 1	27 (29)	1781 (33)		8 (21)	19 (35)		614 (28)	1194 (37)	
Tertile 2	35 (37)	1803 (33)		14 (36)	21 (38)		698 (31)	1140 (35)	
Tertile 3	32 (34)	1791 (33)		17 (44)	15 (27)		905 (41)	918 (28)	
10 km			0.78			< 0.01			< 0.01
Unexposed	406	19,298		259	147		12,158	7546	
Tertile 1	48 (32)	2834 (33)		13 (22)	35 (38)		1109 (31)	1773 (35)	
Tertile 2	48 (32)	2838 (33)		17 (29)	31 (34)		1098 (31)	1788 (35)	
Tertile 3	54 (36)	2830 (33)		29 (49)	25 (27)		1362 (38)	1522 (30)	
Abandoned wells									
10 km			0.77			< 0.01			0.07
Unexposed	494	25,144		298	196		14,755	10,883	
Tertile 1	22 (35)	884 (33)		9 (45)	13 (31)		343 (35)	563 (32)	
Tertile 2	22 (35)	884 (33)		7 (35)	15 (36)		298 (31)	608 (35)	
Tertile 3	18 (29)	888 (33)		4 (20)	14 (33)		331 (34)	575 (33)	

Number of wells	Median (IQR)	Median (IQR)		Median (IQR)	Median (IQR)		Median (IQR)	Median (IQR)	
Active									
5 km	26 (7–76)	20 (5–69)		41 (11–93)	20 (5–45)		30 (6–101)	17 (5–46)	
10 km	85 (37–267)	78 (31–264)		197 (51–362)	56 (33–174)		118 (29–300)	67 (32–224)	
Abandoned									
5 km	5 (2–28)	10 (3–31)		2 (1–17)	5 (2–32)		12 (3–32)	9 (3–30)	
10 km	25 (5–73)	25 (7–75)		22 (5–49)	26 (6–83)		25 (7–74)	24 (7–76)	

### Exposure to active oil and gas wells and Ewing sarcoma risk

**Fig. 2:**
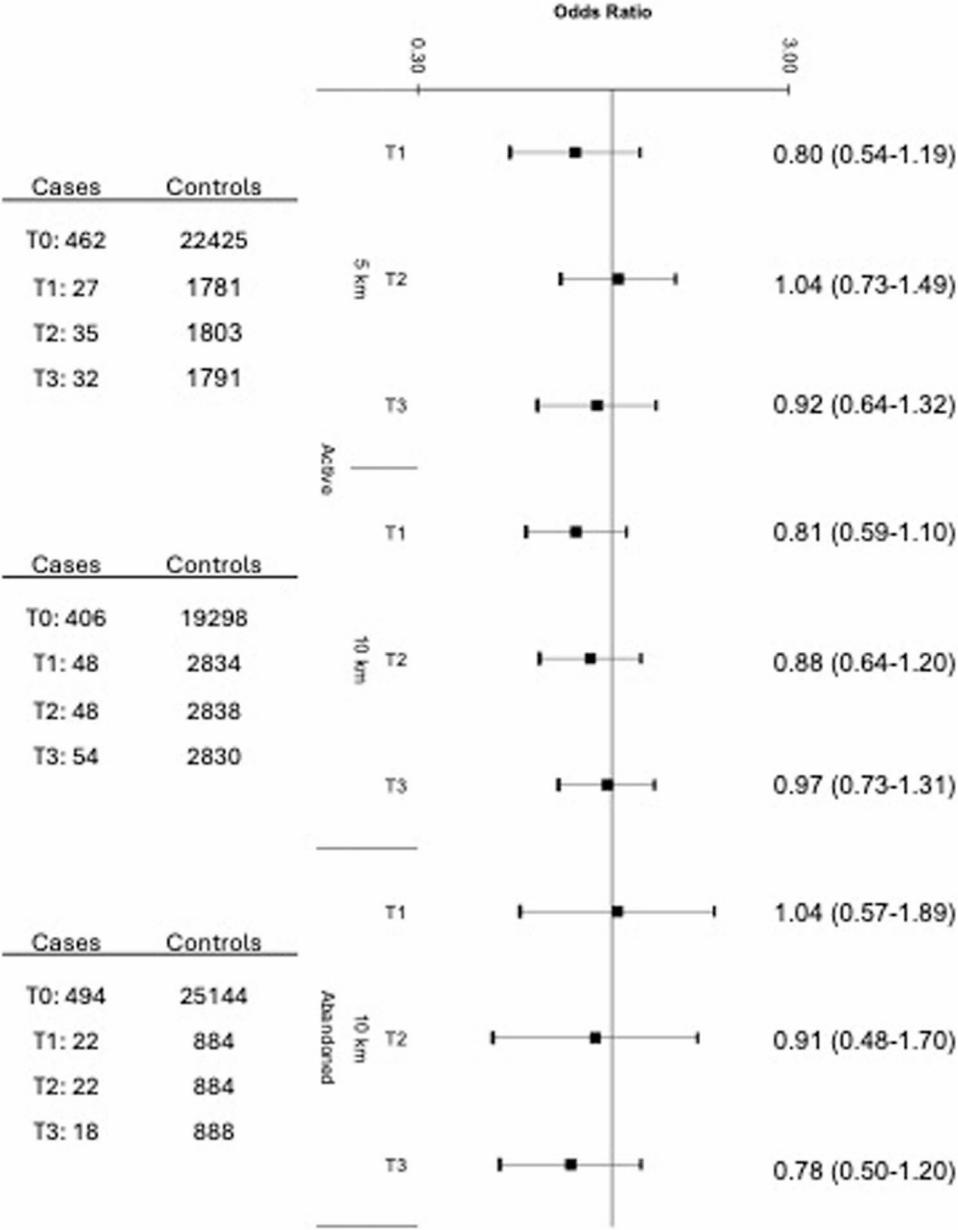
Odds ratios and 95% confidence intervals for the association between OGD exposure to active or abandoned wells (measured by tertiles of intensity) and Ewing sarcoma risk in the overall study population (558 cases and 27,800 controls), as compared to unexposed children (T0). Reference category (T0) for each analysis is unexposed children (0 active or abandoned wells within the designated buffer zone). Odds Ratios and 95% Confidence Intervals were derived from a multivariable logistic regression model that adjusted for birth year, sex, age at diagnosis, race and ethnicity, birth weight, and community-level socio-economic status (i.e., Social Vulnerability Index). Group sample sizes (n) are shown to the left of the corresponding estimates

**Fig. 3:**
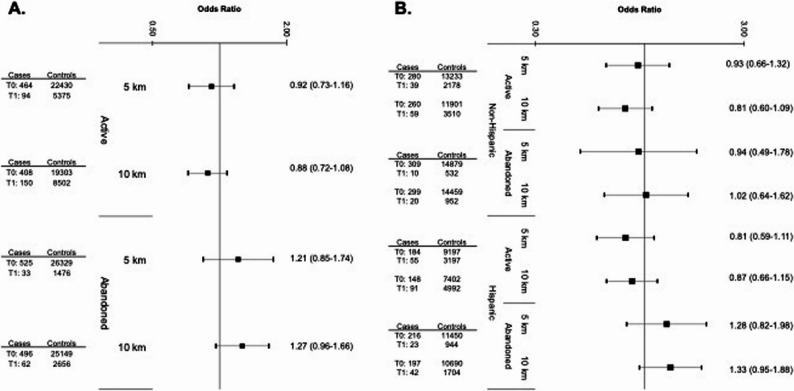
Odds ratios and 95% confidence intervals for the association between OGD exposure to active or abandoned wells (measured dichotomously) and Ewing sarcoma risk in the overall study population (Panel **A**) and stratified by Hispanic ethnicity (Panel **B**), as compared to unexposed children (T0). Reference category (T0) for each analysis is unexposed children (0 active or abandoned wells within the designated buffer zone). Odds Ratios and 95% Confidence Intervals were derived from a multivariable logistic regression model that adjusted for birth year, sex, age at diagnosis, birth weight, and community-level socio-economic status (i.e., Social Vulnerability Index). Group sample sizes (n) are shown to the left of the corresponding estimates

There were no associations between prenatal exposure to active OGD and Ewing sarcoma risk when considered by low, medium, or high intensity (Fig. 2). This remained the case when stratified by Hispanic ethnicity (Supplementary Figure S1) and in sensitivity analyses adjusting for region of birth (Supplementary Figure S2). Among non-Hispanic cases, low-intensity exposure to active OGD within 10 km was associated with a reduction in risk (Tertile 1 OR: 0.51, 95% CI: 0.31–0.96), though this was not consistent for Tertiles 2 or 3. Likewise, exposure to any active OGD (dichotomous measure: yes vs. no) within 10 km was not associated with Ewing sarcoma risk in the total population (OR: 0.88 [95% CI: 0.72–1.08]; Fig. 3, Panel A). Results for active wells remained relatively consistent when stratifying by Hispanic ethnicity (Fig. 3, Panel B).

### Exposure to plugged and abandoned oil and gas wells and Ewing sarcoma risk

Similarly, there were no associations between prenatal exposure to abandoned OGD and Ewing sarcoma risk when considered by low, medium, or high intensity (Fig. 2). However, children with exposure to any abandoned well within 10 km were 1.27 (0.96–1.66) times as likely to develop Ewing sarcoma compared with unexposed children (Fig. 3, Panel A), though the confidence interval was wide. The odds of Ewing sarcoma were also somewhat elevated for children residing within 5 km of abandoned wells (1.21 [0.85–1.74]), though the association did not reach statistical significance. When stratified by ethnicity, the association was similarly elevated among Hispanic children exposed to abandoned OGD within 10 km (1.33 [0.95–1.88]; Fig. 3, Panel B) and absent in non-Hispanic children (1.02 [0.64–1.62]).

### Exposure to all oil and gas wells and Ewing sarcoma risk

**Fig. 4:**
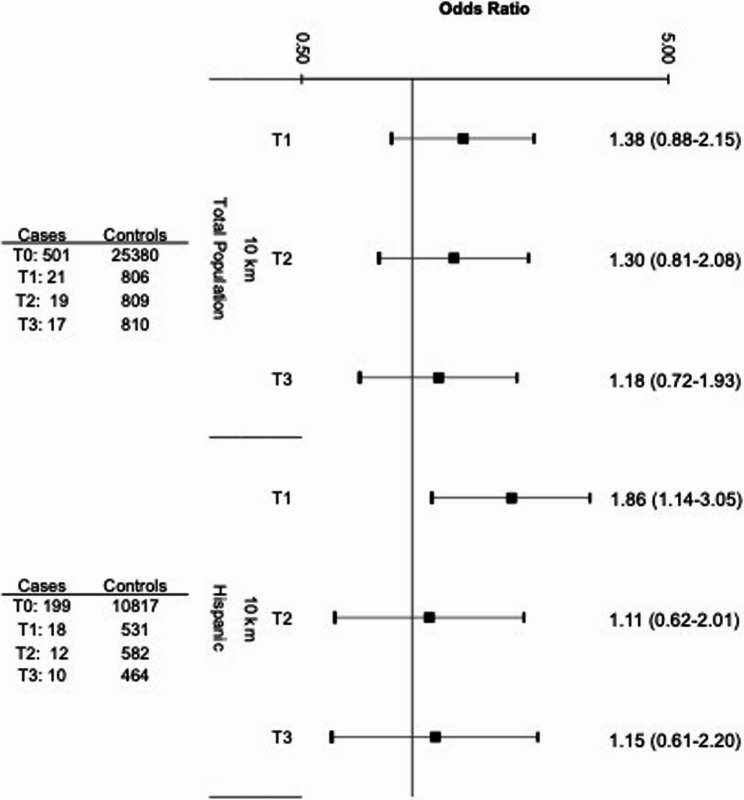
Odds ratios and 95% confidence intervals for the association between OGD exposure to both active and abandoned wells combined (measured by tertiles of intensity) and Ewing sarcoma risk in the overall study population (558 cases and 27,800 controls) and stratified by Hispanic ethnicity as compared to unexposed children (T0). *Reference category (T0) for each analysis is unexposed children (0 active or abandoned wells within the designated buffer zone).* Odds Ratios and 95% Confidence Intervals were derived from a multivariable logistic regression model that adjusted for birth year, sex, age at diagnosis, race and ethnicity, birth weight, and community-level socio-economic status (i.e., Social Vulnerability Index). Group sample sizes (n) are shown to the left of the corresponding estimates. The sample size of the exposed non-Hispanic group was too small to provide informative results

When considering exposure to all wells combined (both active and abandoned, Fig. 4), prenatal residential exposure within 10 km was associated with a modest non-significant elevation in Ewing sarcoma risk in Tertiles 1 (1.38 [0.88–2.15]) and 2 (1.30 [0.81–2.08]), but not Tertile 3 (1.18 [0.72–1.93]). When stratified by Hispanic ethnicity, Hispanic children with low intensity exposure had significantly increased Ewing sarcoma risk (1.86 [1.14–3.05]). This association was not present in the medium and high intensity exposure tertiles.

## Discussion

In our study of 558 California children with Ewing sarcoma and 27,800 birth year-matched controls, our results generally do not indicate an association between prenatal exposure to active OGD and the risk of pediatric Ewing sarcoma. However, we did see suggestively elevated risk associated with exposure to abandoned wells during the prenatal window, particularly among Hispanic children. Hispanic children were also significantly more likely to be exposed to active and abandoned OGD activity than non-Hispanic children.

There is very little literature in this area with which to compare our results. Existing literature consists of a grey literature study of four counties in Pennsylvania and a more recent Pennsylvania report including only 20 cases of Ewing sarcoma [35, 72]. Neither of these studies noted any significant associations between OGD exposure and Ewing sarcoma risk or incidence, though for the latter report the sample size was extremely limited. Additionally, these populations are largely non-Hispanic White, whereas we observed a notable increase in risk among Hispanic children. While we did see elevated risk in Hispanic children exposed to abandoned wells, we did not observe a monotonic increase in risk across increasing tertiles of exposure. This could reflect a small number of exposed cases at the highest tertile, exposure misclassification from a distance-based proxy, or random variation, though these factors do not rule out a true association.

OGD has also been associated with other childhood cancers, mainly hematologic malignancies [33, 34, 37]. However, our observed association between exposure to abandoned wells and Ewing sarcoma risk is plausible. Benzene and other aromatic hydrocarbons emitted by OGD [28] have known clastogenic properties[73–77], and may also cause DNA damage via oxidative stress [78]. Exposure to aromatic hydrocarbons has been associated with chromosomal instability in adult poputions [74]. 

Little is known about the exposures or potential health effects associated with plugged and abandoned wells. One exposure study conducted in California reported that Hispanic and non-Hispanic Black people had disproportionate exposure to abandoned OGD wells [79]. We observed similar exposure disparities among Hispanic children in our study, who were significantly more likely to be exposed to both active and abandoned OGD than non-Hispanic children. Abandoned wells are known to emit both toxic volatile and aromatic compounds as well as greenhouse gases like methane, similarly to active OGD [39–43]. This significant exposure disparity could have broader health implications for California children.

Notably, Ewing sarcoma is not generally thought to have strong environmental risk factors. A study of PM_2.5_ exposure and Ewing sarcoma risk in this same California population also reported elevated risk among Hispanic children [27]. Air pollution is one potential pathway through which OGD could influence children’s health outcomes, and emissions of carcinogenic agents have been documented [31, 80–82]. It is also important to note that the etiology of Ewing sarcoma is not well understood, and as such there may also be unmeasured confounding from unknown sources both in our analysis and in others. Genetic ancestry is one potential source of confounding in this analysis. Ancestry, related to germline susceptibility, is among the strongest risk factors for Ewing sarcoma [83, 84]. Incidence tends to be highest among children with European ancestry and comparatively lower among Hispanic and non-Hispanic Black and Asian children[50, 52, 85, 86]; these patterns are reflected in our population. The reduction in risk among Hispanic children may be more pronounced among those with Native American and Indigenous ancestry. While we adjusted for self-reported race and ethnicity in our analysis, this is often an inadequate proxy for genetic ancestry. This is particularly true among Hispanic and Latinx populations, where self-reported Hispanic ethnicity generally comprises a highly diverse admixed population with variable ancestry components. Notably, socio-economic status is also strongly associated with genetic ancestry [87–89]. However, we suspect that confounding by genetic ancestry would drive our observed association towards the null, meaning the true association may be stronger than reported.

This study has several limitations informing its interpretation. Ewing sarcoma is an extremely rare cancer, with an expected incidence of less than 3 in a million per year among children andadolescents [7, 90]. While our sample size of 558 is impressive for epidemiologic studies of this cancer, it nonetheless limited the type and number of analyses we could perform. Additionally, the quality of the data on abandoned or plugged wells is uncertain. Abandoned wells, by definition, do not tend to have high-quality or consistent reporting information. Because we limited our analysis to those wells with enough information to determine a status of plugged and abandoned, it is likely that the number of abandoned wells included in our dataset underestimates the true number. This also suggests that we may be underestimating exposure to abandoned wells, which may have biased our associations towards the null. We relied on birth address abstracted from the birth record. Using birth residence to assign exposure for a cancer with peak onset in adolescence could introduce exposure misclassification due to unaccounted residential mobility or other factors [91]. Past studies by our group and others on similar spatial exposures have not found residential mobility to be a major source of error for other health outcomes, such as leukemia and birth outcomes, although these generally had shorter latency periods. Moreover, we focused on the potential developmental origins of this disease and exposures occurring during the prenatal window, when the birth address is likely to be most accurate. Increased Ewing sarcoma risk associated with birth weight [50] and hernia [57] both support the potential prenatal origins of this disease, despite a peak incidence in mid-adolescence. Finally, it is possible that our study could be subject to unmeasured confounding by other competing environmental exposures, such as air pollution. However, since air pollution is a potential exposure pathway relevant to OGD, it is impossible to disentangle the potential influence of non-OGD sources.

This study has several notable strengths, including a diverse study population, a long time period for case ascertainment, a large study size for a very rare cancer, and examination of conventional, unconventional, and abandoned/plugged OGD. Selection bias is unlikely to affect our study because this is a registry-based linkage study with no need to contact any subjects for participation. We also minimized the potential for differential misclassification when assigning exposures by masking case/control status.

In this registry-based case-control study, there is some indication that exposure to abandoned wells may be associated with an increase in Ewing sarcoma risk among California children, particularly Hispanic children. We also identified an exposure disparity among Hispanic children, which could have implications for other health outcomes, including other childhood cancers. Further replication in different populations and regions with diverse patterns of OGD activity is necessary to draw concrete conclusions.

## Supplementary Information

Supplementary Material 1.

## Data Availability

No datasets were generated or analysed during the current study.
