# Supplementary material for: Residential proximity to active and abandoned oil and gas development and risk of childhood Ewing sarcoma in California
Source: Environ Health. 2026 Jan 14;25:12. doi: 10.1186/s12940-025-01259-3 (PMC12888232; doi:10.1186/s12940-025-01259-3)
Supplement: Supplementary file 1 — Supplementary Material 1. [file 12940_2025_1259_MOESM1_ESM.docx]

**Supplementary Material for “Exposure to active and abandoned oil and gas development and risk of childhood Ewing sarcoma in California”**

**Supplementary Figure S1.** Odds ratios and 95% confidence intervals for the association between OGD exposure to active or abandoned wells and Ewing sarcoma risk, stratified by Hispanic ethnicity.

*Odds Ratios and 95% Confidence Intervals; adjusted for birth year, sex, age at diagnosis, race and ethnicity, birth weight, and community-level socio-economic status.*

****Supplementary Figure S2.** Odds ratios and 95% confidence intervals for the association between OGD exposure to active or abandoned wells and Ewing sarcoma risk in the total population, adjusted for region of birth.

*Odds Ratios and 95% Confidence Intervals; adjusted for birth year, sex, age at diagnosis, race and ethnicity, birth weight, region of birth, and community-level socio-economic status.*
